# A retrospective analysis of trends in maternal mortality in a Gambian tertiary health centre

**DOI:** 10.1186/s13104-017-2817-0

**Published:** 2017-10-06

**Authors:** Patrick Idoko, Matthew O. Anyanwu, Sabel Bass

**Affiliations:** 1 School of Medical and Allied Health Sciences, University of The Gambia, Banjul, The Gambia; 2 Edward Francis Small Teaching Hospital, Banjul, The Gambia

**Keywords:** Maternal mortality, Gambia, Trend

## Abstract

**Background:**

Maternal mortality ratio (MMR) has been on the decline in the Gambia since 1990. However, there has been no steady decline in maternal mortality ratio in the Edward Francis Small Teaching Hospital, the only tertiary health facility in the Gambia. The aim of the study is to determine the trend in maternal mortality over the last 8 years.A retrospective review of all maternal deaths occurring at the Edward Francis Small Teaching Hospital from 1st January 2007 to 31st December 2014 was done. Case abstraction was done with a pre-structured questionnaire using the WHO definition of maternal mortality.

**Results:**

There were 663 maternal deaths recorded during the study period. During the same period the total number of live births were 38,896. The annual MMR in each year varied with a range between 1461 and 2105 per 100,000 live births. The MMR in the hospital in on the rise compared to earlier studies. The causes of maternal mortality have not changed much in the hospital. However, the seasonal variation in maternal mortality in earlier studies attributed to the influence of malaria and anaemia was not seen in this study. We attribute this change to the widespread use of intermittent prophylactic treatment for malaria in the antenatal period.

**Conclusion:**

While MMR was decreasing in the country, it was increasing in the only tertiary health facility in the country. This was attributed to increasing referrals from other health facilities. The influence of malaria and anemia as a cause of maternal mortality seems to be declining.

## Background

The death of a woman during pregnancy or childbirth is one of the most tragic, painful, unforgettable and often preventable tragedies that can befall a family and community in Africa. Such maternal mortality is one of the main indicators of the quality of health care including health system delivery in a country. It is a reflection of the educational level, economic empowerment and social justice in a community. In 2013, 289,000 women died during and following pregnancy and childbirth [1]. Almost all of these deaths occurred in low-resource settings, and most could have been prevented [1]. The high number of maternal deaths in some areas of the world reflects inequities in access to health services, and highlights the gap between rich and poor.

Improving maternal health is one of the millennium development goals (MDG) adopted by the international community in 2000. The fifth MDG committed countries to reducing maternal mortality by three quarters between 1990 and 2015. Globally, there has been a 45% decline in maternal mortality since 1990 [1]. Several countries in sub-Saharan Africa have halved their maternal mortality ratio (MMR) since 1990 [1]. The Gambia has also seen substantial improvements in MMR [2]. The World Health Organisation (WHO) estimates suggest that MMR has decreased from 730/100,000 live births in 2001 to 430/100,000 by 2012 in the Gambia [2]. However, the WHO figures refer to national estimates. Measuring maternal mortality in developing countries where most maternal deaths occur is challenging for several practical reasons. Maternal deaths may not occur in a health facility, cultural practices surrounding burial rites do not support autopsy and legislation on mandatory maternal death reporting are non-existent and where they exist are not enforced. This invariably means the current approaches to measuring maternal mortalities are not precise, are resource intensive and present often misleading results [3]. Despite this drop in maternal mortality, the Gambia still has one of the highest MMR in sub Saharan Africa [2].

The large majority of Gambian women (86.2%) receive antenatal care from a skilled health professional [4]. This is not dependent on whether the woman resides in an urban or a rural area. However, only 57.2% of all deliveries are conducted by a skilled health professional [4].

Edward Francis Small Teaching Hospital (EFSTH) is the only tertiary health centre in the Gambia. It is the main referral hospital in the country with specialist care (including blood transfusion and surgical services) available whenever needed. These services are provided free to women. Of the 55,969 annual number of births in the Gambia [5], over 5000 (9%) occur in the hospital. However, the hospital accounts for more than 30% of the 340 annual maternal deaths in the Gambia [5]. Previous studies from Royal Victoria Hospital (RVH) now EFSTH found that the MMR of 737/100,000 live births in 1991/1992 had increased to 1121/100,000 live births by 2000/2001 [6, 7]. The number of specialists (obstetricians) providing these services varied between 3 and 5 during the study period. They were assisted by between 3 and 9 physicians (non-specialists) and 22 midwives. The variation in staff strength was usually due to high staff attrition. There is a blood bank in the hospital but blood is often in short supply and patient’s relatives are often required to donate blood for their sick relative. This often results in delay in providing emergency blood transfusion services. Facilities and manpower exist to carry out emergency caesarean sections whenever needed.

This study determined the trend in maternal mortality in the 8-year period from January 2007 to December 2014. The study also identified trends in causes of maternal deaths as well as demographic changes in maternal deaths.

## Methods

A retrospective review of all maternal deaths occurring at the Edward Francis Small Teaching Hospital from 1st January 2007 to 31st December 2014 was done. Case notes of maternal deaths prior to 2007 were not readily available and where available had significant amounts of missing data. Thus, 2007 was chosen arbitrarily as the study start date due to availability of data. All maternal deaths that occurred during the study period were identified from the maternal mortality register of the hospital. A maternal death was defined as death of a woman while pregnant or within 42 days of termination of pregnancy from any cause related to or aggravated by the pregnancy or its management. Pregnant women who died from accidental or intentional injuries were excluded from the study. The case notes of all maternal deaths that met the inclusion criteria were obtained and case abstraction done with a pre-structured questionnaire. The cause of death was assigned based on clinical findings with laboratory support when this was available. Autopsy was not done in any of the maternal deaths. Anaemia was defined as a hemoglobin concentration of < 10 g/dl. The number of live births during the study period was obtained from the labour ward register. MMR was calculated as number of maternal deaths per 100,000 live births. The number of live births and maternal deaths were grouped into two periods to correspond to the malaria season (September–December) and non-malaria season (January–August) in the Gambia. Descriptive statistics were generated using the Epi-Info 7 statistical software from CDC Atlanta.

Approval for the study was obtained from the Ethics Committee, Edward Francis Small Teaching Hospital.

## Results

**Fig. 1:**
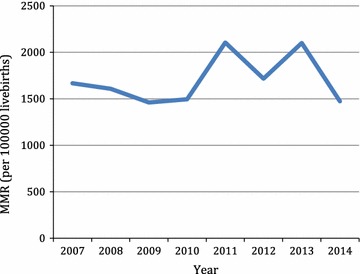
Trends in MMR in EFSTH 2007–2014

There were 663 maternal deaths during the study period. The MMR during the study period was 1705/100,000 live births (Table 1). Seventy-one of the case notes were missing (10.7% attrition). Some factors like marital status, religion, education and occupation could not be assessed as the information were missing in most of the case notes. A fluctuating trend in MMR with 2 peak periods in 2011 and 2013 was observed (Fig. 1).

**Table 1:** Trend in maternal mortality: 2007–2014

	2007–2010	2011–2014	Total
Maternal mortality ratio (MMR)			
Total births	24,080	20,227	44,307
Live births	21,353	17,543	38,896
Maternal deaths	332	331	663
MMR (per 100,000 live births)	1555	1887	1705

	n = 260	n = 330	n = 590
Demographic characteristics of maternal deaths (%)			
Age (years)			
< 20	11.9	11.5	11.7
20–29	45.4	43.9	44.6
30–39	35.8	38.2	37.1
≥ 40	6.9	6.4	6.6

	n = 260	n = 331	n = 591
Parity			
0	26.2	21.8	23.7
1–4	47.3	42.6	44.7
≥ 5	26.5	35.6	31.6

	n = 332	n = 331	n = 663
Antenatal clinic attendance			
Yes	37.3	65.0	51.1
No	1.2	2.7	2.0
Missing	61.4	32.3	46.9

	n = 282	n = 331	n = 613
Residence			
Urban	43.3	49.8	46.8
Rural	56.7	50.2	53.2

	n = 260	n = 330	n = 590
Age (years)			
<20	11.9	11.5	11.7
20–29	45.4	43.9	44.6
30–39	35.8	38.2	37.1
≥ 40	6.9	6.4	6.6

	n = 260	n = 331	n = 591
Parity			
0	26.2	21.8	23.7
1–4	47.3	42.6	44.7
≥ 5	26.5	35.6	31.6

	n = 332	n = 331	n = 663
Antenatal clinic attendance			
Yes	37.3	65.0	51.1
No	1.2	2.7	2.0
Missing	61.4	32.3	46.9

	n = 282	n = 331	n = 613
Residence			
Urban	43.3	49.8	46.8
Rural	56.7	50.2	53.2

	n = 332	n = 331	n = 663
Causes of maternal deaths (%)			
Haemorrhage	22.0	31.2	26.5
Hypertensive	18.4	20.5	19.8
Sepsis	8.1	13.1	10.6
Anaemia	9.3	8.5	8.9
Malaria	2.4	3.0	2.7
Pulmonary embolism	1.5	3.6	2.6
HIV/AIDS	2.2	1.5	1.8
Cardiomyopathies	2.2	4.2	3.2
Liver disease	0.3	1.8	1.1
Anaesthesia	1.5	1.2	1.4
Transfusion reactions	0.6	0.3	0.5
Other medical conditions	0.6	3.3	2.0
Uncertain or not documented	7.8	3.3	6.3
Missing case notes	21.6	0.0	10.8
Dead on arrival	1.5	4.5	3.0
Total	100.0	100.0	100.0

	n = 332	n = 331	
Timing of death in relation to pregnancy (%)			
Antepartum death	16.9	10.0	13.4
Postpartum death	61.4	84.6	73.0
Missing	21.7	5.4	13.6
Total	100.0	100.0	100.0

	n = 258	n = 322	n = 580
Duration of hospitalization at EFSTH before death (%)			
< 24 h	40.3	52.2	46.9
24–48 h	15.1	18.3	16.9
49–72 h	14.0	6.2	9.7
3–7 days	14.7	13.7	14.1
> 7 days	15.9	9.6	12.4
Total	100.0	100.0	100.0

**Fig. 2:**
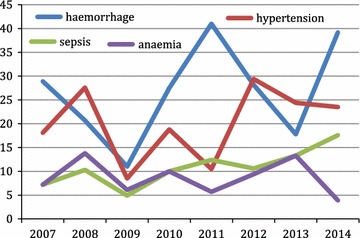
Trend in common causes of maternal mortality 2007–2014

Haemorrhage, hypertensive disease, sepsis and anaemia were consistently the commonest causes of maternal mortality (Table 1). The common causes of maternal mortality were fluctuating but sepsis seems to be rising (Fig. 2).

More deaths occurred in the postpartum period and this trend was rising from 61 to 85% (Table 1). Majority of deaths occurred within 24 h of arrival to the hospital. The trend showed an increase from 40 to 52% (Table 1).

**Fig. 3:**
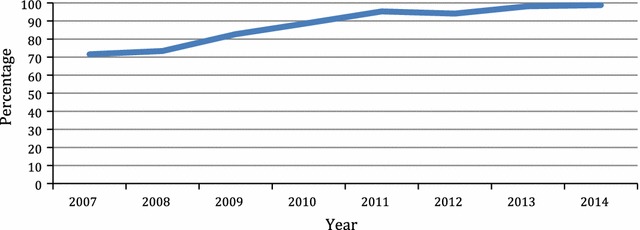
Proportion of maternal mortality referred from other health facilities (percentage)

Over the years, the proportion of maternal deaths referred from other health facilities has been on a steady increase (Fig. 3).

**Fig. 4:**
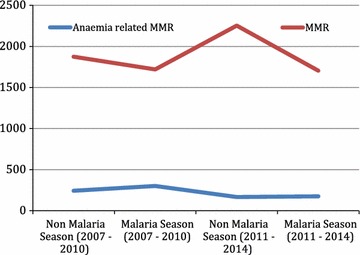
Trends in MMR and anaemia-related MMR between malaria and non-malaria season 2007–2014

The trends in MMR and anaemia-related MMR between malaria (September–December) and non-malaria season (January–August) 2007–2014 is shown in Table 2 and Fig. 4. Anaemia related deaths were higher during the malaria season but not high enough to cause a greater burden on maternal mortality.

**Table 2:** Trend in seasonal variation in MMR at EFSTH 2007–2014

	2007–2010			2011–2014		
	January–August Number (%)	September–December Number (%)	Relative risk^a^	January–August Number (%)	September–December Number (%)	Relative risk^a^
Live births	13,667 (64.0)	7686 (36.0)	0.8710*	11,263 (64.2)	6280 (35.8)	0.7367**
Maternal deaths	223 (67.2)	109 (32.8)		235 (71.0)	96 (29.0)	
MMR	1631.7	1418.2		2086.5	1528.7	

* p = 0.2338, ** p = 0.1110

^a^Relative risk = September–December/January–August (95% confidence interval)

## Discussion

There were 663 maternal deaths recorded during the study period-January 2007 to December 2014. During the same period the total number of deliveries were 44,307 however, 38,896 were live births. The estimated maternal mortality ratio (MMR) in each year varied with a range between 1461 and 2105 per 100,000 live births. A study conducted in the same hospital in the 1990s had MMR estimated at 736 per 100,000 live births [6]. In our study the lowest MMR was 1461 per 100,000 live births (2009) which signify a twofold increase from the previous study [6]. Also, the study revealed, approximately a 45% rise of MMR between 2009 and 2011. Similar rise occurred between 2012 and 2013. The MMR between 2007–2010 and 2011–2014 was 1555 and 1887 per 100,000 live births respectively, which showed about 20% increase. Therefore, the trend overall of MMR at EFSTH is on the increase despite paradoxical decrease in the national MMR [4]. The proportion of women who have antenatal care and the proportion of deliveries attended by skilled birth attendants have increased over the years in the Gambia [8–10]. EFSTH as the only tertiary health centre is thus getting more cases that would hitherto not have presented to any health facility. This can be deduced from the fact that the proportion of maternal deaths that were referred from other health facilities has been steadily increasing (Fig. 3). The other contributory factor to this trend is delayed referral of cases. Our study showed that the proportion of maternal deaths that occurred within 24 h of admission at EFSTH had increased from 40% in 2007–2010 to over 52% between 2011–2014 (Table 1). Increasing referrals from other health facilities and late presentation may explain the rising trend in MMR in EFSTH. However, similar studies from other tertiary hospitals also showed fluctuating trend in MMR [11–14].

The impact of demographic characteristics of maternal deaths in this 8-year review also had remarkable findings. A study by Asamoah et al. [11] in Ghana and another in India [15] showed that majority of women who died were from rural areas and did not attend antenatal clinic. However, this was not the case in our study where 51.1% of mortalities were women who had antenatal care. This scenario is not common in literatures regarding maternal mortality. The Gambia has a wide coverage of antenatal care; over 90% receive care at least once during pregnancy [4]. Healthcare services are provided free to all pregnant women in the Gambia. This is a huge financial burden for a country with a GDP of $800 per capita [16]. The dwindling economic fortune in recent years [16] will inadvertently affect the quality of healthcare available to pregnant women. Hence, although over 90% of pregnant Gambian women have some form of antenatal care, our findings reflect the quality of the maternity services and referral system in the Gambia.

In our study haemorrhage (26.5%), hypertensive disease (19.8%), sepsis (10.6%) and anaemia (8.9%) were consistently the commonest causes of maternal mortality in the period under review. Obstetrics haemorrhage remained the commonest cause (26.5%) and advancing maternal age and parity has strongly been associated with maternal deaths. Advanced maternal age and increasing parity are independent risk factors of obstetrics haemorrhage. Previous studies conducted in the Gambia on maternal mortality in rural and tertiary hospitals revealed haemorrhage as the consistent commonest cause of maternal death [6, 17–19]. The trend has not changed for over 3 decades. The reasons have not changed remarkably either, as late arrival to hospital due to delay in decision and transport still persists.

In this review, 46.9% of deaths occurred within 24 h of arrival. Also availability of compatible blood and donors has remained a challenge. In our study 73% of deaths occurred in the postpartum period and haemorrhage remained the consistent commonest cause. This is consistent with similar studies conducted in the sub-region [11, 20–22].

While our study shows similar pattern on the causes of mortality from global studies [23–26], it is pertinent to note that studies from developed countries highlight indirect causes of maternal deaths like cardiac diseases as being more common [27–29]. Direct causes of maternal death like haemorrhage, hypertension and sepsis are largely preventable. That they continue to feature as major causes of maternal deaths in the Gambia is a reflection of the socioeconomic development of the country. Blood, anti-hypertensive medications and antibiotics may not be available when needed as a consequence of this economic challenge.

In our study anaemia is the most common cause of indirect maternal death however, the impact of malaria in pregnancy on chronic anaemia may have reduced as malaria contributed approximately 3% of maternal deaths in the period under review. A previous study conducted in the same hospital showed that during the malaria season, there was a 168% increase in the maternal mortality ratio (MMR), a threefold increase in the proportion of deaths due to anaemia, and an eightfold increase in the anaemia MMR [7]. In comparison, this study found a steady decline in MMR during the malaria season although not statistically significant (Table 2, Fig. 3). Figure 3 also shows that the anaemia related maternal mortality has declined steadily over the years. The proportion of women using sulphadoxine–pyrimethamine combination for intermittent preventive treatment of malaria in pregnancy has steadily improved from 21% in 2007 to more than 90% in 2010 [9, 10]. This may explain the decreased role of anaemia and malaria as causes of maternal mortality. This may also explain why the seasonal variation in maternal mortality seen in a previous study was not seen in this study [7]. However, other studies from the sub-region also point to anaemia as a leading cause of indirect maternal deaths [21, 30].

The study has a number of limitations including the proportion of missing case notes (10.7%) and missing information from available case notes. In addition, autopsy is not routinely done in this environment and thus the causes of maternal death were determined clinically in this study.

## Conclusions

In conclusion, we found that MMR was increasing in EFSTH while the MMR was decreasing in the country. This is most likely due to increasing use of healthcare facilities leading to more referrals and late presentation to the hospital. We also noted that the effect of malaria (and anaemia) on MMR seems to be decreasing since the introduction of intermittent prophylactic treatment for malaria in pregnancy. There is a need to improve the quality of care in primary and secondary health facilities to enable them recognize and transfer high-risk obstetric cases in good time. Efforts need to be intensified to ensure adequate supply of blood, anti-hypertensive drugs (including magnesium sulphate) and antibiotics as well.

## Abbreviations

CDC: Centre for Disease and Control
EFSTH: Edward Francis Small Teaching Hospital
MDG: millennium development goals
MMR: maternal mortality ratio
RVH: Royal Victoria Hospital
WHO: World Health Organization
